# Supplementary material for: Absolute protein quantification using fluorescence measurements with FPCountR
Source: Nat Commun. 2022 Nov 3;13:6600. doi: 10.1038/s41467-022-34232-6 (PMC9633595; doi:10.1038/s41467-022-34232-6)
Supplement: Supplementary file 2 — Reporting Summary [file 41467_2022_34232_MOESM2_ESM.pdf]

nature portfolio

Corresponding author(s): Eszter Csibra and Guy-Bart Stan

Last updated by author(s): October 11, 2022

Reporting Summary

Nature Portfolio wishes to improve the reproducibility of the work that we publish. This form provides structure for consistency and transparency in reporting. For further information on Nature Portfolio policies, see our Editorial Policies and the Editorial Policy Checklist.

Statistics

For all statistical analyses, confirm that the following items are present in the figure legend, table legend, main text, or Methods section.

n/a	Confirmed	
☐	☒	The exact sample size (*n*) for each experimental group/condition, given as a discrete number and unit of measurement
☐	☒	A statement on whether measurements were taken from distinct samples or whether the same sample was measured repeatedly
☒	☐	The statistical test(s) used AND whether they are one- or two-sided<br>*Only common tests should be described solely by name; describe more complex techniques in the Methods section.*
☒	☐	A description of all covariates tested
☐	☒	A description of any assumptions or corrections, such as tests of normality and adjustment for multiple comparisons
☐	☒	A full description of the statistical parameters including central tendency (e.g. means) or other basic estimates (e.g. regression coefficient) AND variation (e.g. standard deviation) or associated estimates of uncertainty (e.g. confidence intervals)
☒	☐	For null hypothesis testing, the test statistic (e.g. *F*, *t*, *r*) with confidence intervals, effect sizes, degrees of freedom and *P* value noted<br>*Give P values as exact values whenever suitable.*
☒	☐	For Bayesian analysis, information on the choice of priors and Markov chain Monte Carlo settings
☒	☐	For hierarchical and complex designs, identification of the appropriate level for tests and full reporting of outcomes
☒	☐	Estimates of effect sizes (e.g. Cohen's *d*, Pearson's *r*), indicating how they were calculated

Our web collection on statistics for biologists contains articles on many of the points above.

Software and code

Policy information about availability of computer code

Data collection	Tecan Spark plate reader software: SparkControl Magellan V 3.1; BMG LabTech CLARIOstar plate reader software: CLARIOstar v5.60 and MARS v3.40.
Data analysis	R v4.0.3, flopR v0.2.0 (https://github.com/ucl-cssb/flopr), fpcountr v0.1.0 (https://github.com/ec363/fpcountr)

For manuscripts utilizing custom algorithms or software that are central to the research but not yet described in published literature, software must be made available to editors and reviewers. We strongly encourage code deposition in a community repository (e.g. GitHub). See the Nature Portfolio guidelines for submitting code & software for further information.

Data

Policy information about availability of data

All manuscripts must include a data availability statement. This statement should provide the following information, where applicable:

- Accession codes, unique identifiers, or web links for publicly available datasets
- A description of any restrictions on data availability
- For clinical datasets or third party data, please ensure that the statement adheres to our policy

This study includes no data deposited in external repositories. Key datasets are provided in the Supplementary Tables. Further datasets are provided in the Source Data file provided with this paper. External data from FPbase was taken from https://www.fpbase.org/.

Field-specific reporting

Please select the one below that is the best fit for your research. If you are not sure, read the appropriate sections before making your selection.

☒ Life sciences ☐ Behavioural & social sciences ☐ Ecological, evolutionary & environmental sciences

For a reference copy of the document with all sections, see nature.com/documents/nr-reporting-summary-flat.pdf

Life sciences study design

All studies must disclose on these points even when the disclosure is negative.

Sample size	No sample size calculations were performed. This wasn't relevant as the aim of these experiments was not to test for a significant difference between populations but rather to test the performance of each method/protocol.
Data exclusions	Data exclusions were not relevant except for exclusion of certain samples using chemical lysis (Fig3E) detailed in the figure legend. These were excluded due to overt scatter identified by very high levels of baseline absorbance.
Replication	Replications were carried out and are presented in the figures.
Randomization	Randomisation was not performed. This wasn't relevant as there are no known steps in the protocols performed that are known to be biased without randomisation.
Blinding	Blinding was not performed. This was not relevant as the conclusions were not gained from subjective assessments of the data, but from a standardised analytical workflow.

Reporting for specific materials, systems and methods

We require information from authors about some types of materials, experimental systems and methods used in many studies. Here, indicate whether each material, system or method listed is relevant to your study. If you are not sure if a list item applies to your research, read the appropriate section before selecting a response.

Materials & experimental systems

n/a	Involved in the study	
☒	☐	Antibodies
☒	☐	Eukaryotic cell lines
☒	☐	Palaeontology and archaeology
☒	☐	Animals and other organisms
☒	☐	Human research participants
☒	☐	Clinical data
☒	☐	Dual use research of concern

Methods

n/a	Involved in the study	
☒	☐	ChIP-seq
☒	☐	Flow cytometry
☒	☐	MRI-based neuroimaging
