# Supplementary material for: Absolute protein quantification using fluorescence measurements with FPCountR
Source: Nat Commun. 2022 Nov 3;13:6600. doi: 10.1038/s41467-022-34232-6 (PMC9633595; doi:10.1038/s41467-022-34232-6)
Supplement: Supplementary file 3 — Description of Additional Supplementary Files [file 41467_2022_34232_MOESM3_ESM.pdf]

Title: Supplementary Data File 1
Description: Comparison of commercially available GFPs

Title: Supplementary Data File 2
Description: Light absorbance of E coli

Title: Supplementary Data File 3
Description: Sequences
